# Bayesian estimation of scaled mutation rate under the coalescent: a sequential Monte Carlo approach

**DOI:** 10.1186/s12859-017-1948-6

**Published:** 2017-12-08

**Authors:** Oyetunji E. Ogundijo, Xiaodong Wang

**Affiliations:** Department of Electrical Engineering, Columbia University, New York, 10027 USA

**Keywords:** Coalescent, Sequential Monte Carlo, Genealogy, Bayesian

## Abstract

**Background:**

Samples of molecular sequence data of a locus obtained from random individuals in a population are often related by an unknown genealogy. More importantly, population genetics parameters, for instance, the scaled population mutation rate *Θ*=4*N*_*e*_ *μ* for diploids or *Θ*=2*N*_*e*_ *μ* for haploids (where *N*_*e*_ is the effective population size and *μ* is the mutation rate per site per generation), which explains some of the evolutionary history and past qualities of the population that the samples are obtained from, is of significant interest.

**Results:**

In this paper, we present the evolution of sequence data in a Bayesian framework and the approximation of the posterior distributions of the unknown parameters of the model, which include *Θ* via the sequential Monte Carlo (SMC) samplers for static models. Specifically, we approximate the posterior distributions of the unknown parameters with a set of weighted samples i.e., the set of highly probable genealogies out of the infinite set of possible genealogies that describe the sampled sequences. The proposed SMC algorithm is evaluated on simulated DNA sequence datasets under different mutational models and real biological sequences. In terms of the accuracy of the estimates, the proposed SMC method shows a comparable and sometimes, better performance than the state-of-the-art MCMC algorithms.

**Conclusions:**

We showed that the SMC algorithm for static model is a promising alternative to the state-of-the-art approach for simulating from the posterior distributions of population genetics parameters.

**Electronic supplementary material:**

The online version of this article (doi:10.1186/s12859-017-1948-6) contains supplementary material, which is available to authorized users.

## Background

Samples of molecular data, such as DNA sequence, taken from a population are often related by an unknown genealogy [1], a family tree which depicts the ancestors and descendants of individuals in the sample and whose shape is altered by the population processes, such as migration, genetic drift, change of population size, etc. [2]. The genetic events and the past history of such population can be studied by estimating the underlying population parameters based on the samples of molecular data from the population [3].

Oftentimes, biologists are interested in an accurate estimation of the population parameters from samples of molecular data because these parameters provide answers to several unanswered biologically motivated questions and sometimes, the knowledge results in new discoveries [4, 5]. For instance, in [6, 7], estimates of some of the population parameters revealed the role of historical processes in the evolution of a population and as well, aided the understanding of microevolutionary processes and lineage divergence through phylogeographical analysis. Further, based on the estimation of these important parameters, [8, 9] were able to infer past environmental conditions (in combination with documented geologic events) that explain the current patterns in the population; they also investigated the role of environmental factors in shaping the contemporary phylogeographic pattern and studied the genetic homogeneity of organisms. Moreover, in species classification, knowledge of these parameters has helped in classifying previously unclassified or wrongly classified organisms [10] and also in investigating the contribution of geographic barriers in the diversification and classification of organisms [11, 12].

In the literature, some methods have been proposed to estimate these important parameters from samples of molecular data from the population of interest. For instance, summary statistics of sample sequences such as Watterson’s theta $\hat {\Theta }_{W}$ [13] can be used to make a fast estimate of *Θ*. However, summary statistics from the molecular data often fail to account for the presence of multiple evolutionary forces [14]. Another approach involves an estimation of the underlying genealogy that represents the individuals sampled from the population and then using this as the basis for parameter estimation [15]. Kuhner [14] noted that except in a few cases of artificially manipulated populations, the exact genealogy of a sample is generally unknown.

Other approaches such as the approximate Bayesian computation (ABC) [16, 17] have been proposed, which are often employed when the likelihood function can not be evaluated. However, a more universal and effective approach to estimating population parameters is the coalescent genealogy sampling method, our focus in this paper [18–21]. Here, the assumption is that the genealogical structure of samples of molecular data from the population is completely unknown, which is a reasonable assumption. Since it is generally impossible to consider all the infinitely large possible genealogies that describe the sampled sequences, coalescent genealogy sampling methods take samples from the posterior distribution of the genealogy (i.e., sampling the more probable ancestral patterns from the infinite set of all possible patterns). In estimating population parameters with the coalescent samplers, two distinct approaches have been proposed: (i) MCMC [18–21] and (ii) importance sampling (IS) [22, 23]. The former is suitable for either a likelihood-based estimation [21] or full Bayesian estimation [20, 21]. However, for the latter, [23] assumes an infinite-sites mutational model which holds an assumption that no site has mutated more than once and thus, this makes it difficult to incorporate less restrictive mutational models [14]. In [22], although there is a slight loss of accuracy in parameter estimation, there is a significant reduction in computational time and a reduction in variance. However, for the [24] noted that MCMC-approach to Bayesian posterior approximation often suffer from two major drawbacks: (i) difficulty in assessing when the Markov chain has reached its stationary regime of interest, and (ii) if the target distribution is highly multi-modal, MCMC algorithms can easily become trapped in local modes. Recently, [25] developed a particle marginal Metropolis-Hastings (PMMH) algorithm that employs a sequential Monte Carlo (SMC) sampler, which has been employed in other areas of computational biology for parameter estimation in Bayesian settings [26, 27], but the genealogy of the observed sequence is assumed known.

In this paper, assuming that the genealogy of the observed sequences is unknown, we present a sequential Monte Carlo (SMC) sampler for static models [24, 28, 29] to search for the highly probable genealogies from the infinite set of all possible genealogies that can describe the observed genetic data, i.e., highly probable samples from the posterior distributions of the genealogy, and other unknown parameters, resulting in a more reliable and accurate estimation of the parameter of interest, *Θ*. We model the observed genetic data using a Bayesian framework and subsequently treat the parameter *Θ*, the genealogy relating the observed data and other mutational model parameters as the unknown parameters of the model. Bayesian inference is an important area in the analyses of biological data [30–34] as it provides a complete picture of the uncertainty in the estimation of the unknown parameters of a model given the data and the prior distributions for all the unknown model parameters. Specifically, we use the SMC method to simulate and approximate, in an efficient way, the joint posterior distribution of *Θ*, the genealogy and other unknown model parameters, by a set of weighted samples (particles) from which the point estimate of *Θ* can be made [24]. SMC is a class of sampling algorithms which combine importance sampling and resampling [35, 36]. When the data generating model is dynamic, one attempts to compute, in the most flexible way, the posterior probability density function (PDF) of the state every time a measurement is received, i.e., data are being processed sequentially rather than as a batch [37–42]. However, in static models, which is the main focus here, the SMC framework for the dynamic model is slightly modified [24, 28, 29] as this involves the construction of a sequence of artificial distributions on spaces of increasing dimensions. This sequence of artificial distributions, however, admits the probability distributions of interest as marginals. As a matter of fact, this procedure is quite similar to the sequential importance sampling (resampling) (SIS) procedure for dynamic models [35] with the only difference being the framework under which the samples are propagated sequentially which results in differences in the calculation of the weights. With the SMC methods, we can treat, in a principled way, any type of probability distribution, nonlinearity and non-stationarity [43, 44]. The algorithms are easy to implement and applicable to very general settings. In addition, in big data analyses, SMC algorithms can be parallelized to reduce the computational time.

Although, the proposed algorithm can be adapted to the likelihood-based framework, we have concentrated on the full Bayesian analysis where we are able to generate highly probable samples from the joint posterior distribution of the genealogy, *Θ*, and other unknown parameters in the model from sample sequences from a population [45]. We compare the proposed method with some existing coalescent-based methods for estimating *θ* [18–21] that rely on the Metropolis-Hastings MCMC (MH-MCMC) algorithms. In terms of the accuracy of the estimates of *Θ*, the proposed SMC method demonstrates a comparable, and sometimes better performance.

The remainder of this paper is organized as follows. In Method section, we describe the system model, problem formulation, the SMC samplers for Bayesian inference, and present the proposed algorithms for estimating *Θ* from molecular data. In Sequential Monte Carlo samplers section, we investigate the performance of the proposed method using simulated datasets obtained from the simulators: *ms* [46] and *Seq-Gen* [47] and also on real biological sequence data from [48], a sequence data that has been extensively used to evaluate the performance of coalescent sampling algorithms. Finally, Results section concludes the paper.

In this paper, we use the following notations:

1. *p*(·) and *p*(·|·) denote a probability and a conditional probability density functions, respectively.
2. *P* *r*(·|·) denotes a conditional probability mass function.
3. $\mathcal {U}(a,b)$ denotes a uniform distribution over the interval [*a*,*b*].

## Method

### System model and problem formulation

**Fig. 1:**
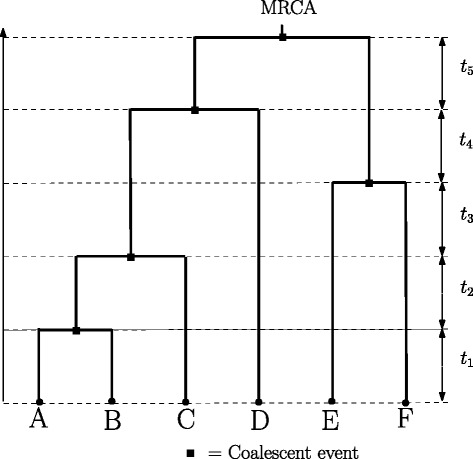
Coalescence. A realization of the coalescent for a sample size of 6. For example, *t*_1_ is the length of the interval during which the genealogy has 6 lineages

Sequence data from random sample of individuals from a population, usually denoted as an *m*×*l* matrix **D** of characters, where *m* denotes the number of sequences and *l* denotes the length of the aligned sequences are often related by an unknown tree or genealogy. For instance, Fig. 1 shows the genealogy representing the relationship between a set of gene copies randomly chosen from a population at the present time and the *coalescent theory* [49–51] describes the distribution of such an unknown genealogy. Specifically, the coalescent is a model that predicts the probability of possible patterns of genealogical branching, working backward in time from the present to the point of a single common ancestor in the past, often referred to as the *most recent common ancestor* (MRCA) as shown in Fig. 1. The probability distribution is given as a product of exponential densities:

1 $$ p(\Upsilon^{'}|N_{e}) = \prod\limits_{k = 2}^{m} \frac{2}{4N_{e}} \exp \left\lbrace \frac{k(k-1)}{4N_{e}}t_{k} \right\rbrace,  $$

where *m* denotes the number of randomly sampled sequences, *N*_*e*_ denotes the *effective population size* and *t*_*k*_ denotes the length of the interval during which the genealogy $\phantom {\dot {i}\!}\Upsilon ^{'}$ has a total of *k* lineages. Since we can not directly observe the coalescence intervals *t*_*k*_, these intervals are often rescaled to the per-site neutral mutation rate *μ*. Hence, *t*_*k*_ in (1) is replaced by *d*_*k*_=*μ* *t*_*k*_ and (1) can be rewritten as [2]:

2 $$ p(\Upsilon|\Theta) = \prod\limits_{k = 2}^{m} \frac{2}{\Theta} \exp \left\lbrace \frac{k(k-1)}{\Theta}d_{k} \right\rbrace  $$

where *Θ*=4*N*_*e*_ *μ* denotes the scaled mutation rate *per site* per generation, which is the parameter of interest to be estimated (note: we have chosen *Θ* instead of *θ* because *θ* is often used to denote the mutation rate *per locus* per generation in related studies).

According to [52], the likelihood function for a given value of *Θ* is given by:

3 $$ \begin{aligned} {}L(\Theta|\mathbf{D}) \,=\,\! Pr(\mathbf{D}|\Theta) &\,=\, \iint Pr(\mathbf{D},\Upsilon,\boldsymbol{\lambda} | \Theta) d\Upsilon d\boldsymbol{\lambda} \\ & \,=\, \iint \!\!p(\boldsymbol{{\lambda}}|\! \Theta) p(\Upsilon| \boldsymbol{\lambda},\! \Theta\!) Pr(\mathbf{D}| \Upsilon,\! \boldsymbol{\lambda},\! \Theta\!) d\Upsilon\! d\boldsymbol{\lambda} \\ & \,=\, \iint p(\boldsymbol{\lambda})p(\Upsilon|\Theta) Pr(\mathbf{D}|\Upsilon, \boldsymbol{\lambda}) d\Upsilon d\boldsymbol{\lambda} \end{aligned}  $$

where *p*(*Υ*|*Θ*) denotes the probability of genealogy given the parameter *Θ*, explicitly stated in (2) (given *Θ*, *Υ* is independent of ***λ***), ***λ*** denotes the parameters of the mutational model, and *P* *r*(**D**|*Υ*,***λ***) denotes the probability of the sequence data **D**, given the genealogy *Υ* and the mutational model [53]. Although, in the analysis of genetic data, different mutational models can be employed, we consider, for the nucleotide sequence datasets, the two-parameter model K80 [54] and the F84 [55] models (the finite-sites models that account for the fact that same site may experience mutation more than once). The former assumes equal nucleotide frequencies among the four nucleotides (i.e., *π*_*A*_=*π*_*C*_=*π*_*G*_=*π*_*T*_=0.25) with an unknown *transition-transversion ratio*, *κ*, while the latter assumes neither the nucleotide frequencies, $\lbrace \pi _{i}: i \in A,C,G,T, \pi _{i} \geq 0, \sum \pi _{i} = 1 \rbrace $, nor *κ* is known. The set of all the mutational model parameters is denoted by ***λ***. (Detailed discussions of the mutational models are given in the Additional file 1).

The goal of the inference is to obtain an estimate of the unknown parameter *Θ* in (2) and (3). To do this, we define a model that generates the sequence data **D** given all the parameters; define suitable prior distributions for all the unknown model parameters, derive the sequence of target distributions for all the parameters, present the SMC algorithm that estimates, in an efficient manner, the joint posterior distribution of all the unknown model parameters, marginalizes out the nuisance/uninteresting parameters and finally, approximates the posterior distribution of parameter *Θ* by a set of weighted samples.

#### Likelihood function

The probability of the observed sequence data **D** given the parameter *Θ* is given explicitly in (3) by [52]. All the elements in (3) except for *P* *r*(**D**|*Υ*,***λ***) have explicit expressions, but *P* *r*(**D**|*Υ*,***λ***) can easily be computed by the procedures highlighted in [53]. Fortunately, an explicit expression for *P* *r*(**D**|*Υ*,***λ***) is not required in the proposed algorithm, as we only need to evaluate it.

#### Prior densities for all model parameters

Here, we discuss the suitable choice of prior distributions for *Θ*, the parameter of interest; the set of unknown parameters of the mutational model, ***λ*** and the genealogy of sampled sequences.

##### Prior density of *Θ*:

We impose a uniform distribution in an interval between 0 and *Θ*_max_, i.e., $\Theta \sim \mathcal {U}(0,\Theta _{\max })$. *Θ*_max_ can be chosen based on some prior biological knowledge that is held about the population. For our experiments, we discuss how this is chosen in the Additional file 1.

##### Prior densities of the mutational model parameters (***λ***):

***λ*** is the set of all the unknown parameters of the mutational model such as the *transition-transversion ratio*, *κ*, and the nucleotide frequencies $\lbrace \pi _{i}: i \in A,C,G,T, \pi _{i} \geq 0, \sum \pi _{i} = 1 \rbrace $. Similar to *Θ*, we impose a uniform distribution on *κ* i.e., $\kappa \sim \mathcal {U}(0,\kappa _{\max })$. The natural choice for the prior distribution of the nucleotide frequency, ***π*** is the Dirichlet distribution i.e. ***π***∼*D* *i* *r*(***α***). The possible choices of *κ*_max_ and ***α***, the concentration parameter of the Dirichlet distribution, are discussed in the Additional file 1.

##### Prior density of the genealogy (*Υ*):

The prior distribution for the genealogy *Υ* is given in (2) and the procedure for simulating a random genealogy from this particular distribution is highlighted in the Additional file 1.

#### Posterior distribution

Given the prior distribution of *Θ*, *p*(*Θ*) and the likelihood function in (3), using Bayes theorem, the posterior distribution of *Θ* is defined as follows:

4 $$ p(\Theta|\mathbf{D}) = \frac{\iint p(\boldsymbol{\lambda}) p(\Theta) p(\Upsilon|\Theta) Pr(\mathbf{D}|\Upsilon,\boldsymbol{\lambda}) d\Upsilon d \boldsymbol{\lambda}}{Z}  $$

where $Z = \iiint p(\boldsymbol {\lambda }) p(\Theta) p(\Upsilon |\Theta) Pr(\mathbf {D}|\Upsilon, \boldsymbol {\lambda }) d\Upsilon d\Theta d \boldsymbol {\lambda }$ is a constant with respect to *Υ*, *Θ* and ***λ***; *p*(***λ***) denotes the prior distribution(s) of the mutational model parameter(s); *p*(*Υ*|*Θ*) denotes the prior distribution of the genealogy, given in (2) and *P* *r*(**D**|*Υ*,***λ***) in (3) denotes the probability of the sequence data, **D** given the genealogy and a mutational model, ***λ***. Although, the marginal posterior distribution of *Θ* has been described in (4), the associated integrals cannot be computed analytically. As a result, we write an expression for the joint posterior distribution of *Θ*, *Υ* and ***λ*** to get rid of the integral in the numerator of (4) as follows:

5 $$ p(\Theta, \boldsymbol{\lambda}, \Upsilon |\mathbf{D}) = \frac{p(\boldsymbol{\lambda}) p(\Theta) p(\Upsilon|\Theta) Pr(\mathbf{D}|\Upsilon, \boldsymbol{\lambda})}{Z},  $$

the denominator in (5) remains a constant. In the new expression for the joint posterior distribution, *p*(***λ***), *p*(*Θ*) and *p*(*Υ*|*Θ*) are the prior distributions of ***λ***, *Θ* and *Υ*, respectively and *P* *r*(**D**|*Υ*,***λ***) is the ‘likelihood function’. It is quite easy to obtain samples from the prior distributions and more importantly, *P* *r*(**D**|*Υ*,***λ***) can be evaluated.

### Sequential Monte Carlo samplers

#### General principle of SMC

Before we introduce the SMC algorithm for the estimation of *Θ*, we will succinctly introduce the general principle of SMC samplers [24, 28, 29, 56, 57] for estimating parameters in static models. Let $\boldsymbol {\mathcal {H}} = [\Theta, \lambda, \Upsilon ]$, then (5) can be re-written as follows:

6 $$ p(\mathcal{\boldsymbol{\mathcal{H}}}|\mathbf{\mathbf{D}}) = \frac{p(\boldsymbol{\mathcal{H}})p(\mathbf{\mathbf{D}}|\boldsymbol{\mathcal{H}})}{Z}  $$

where $p(\boldsymbol {\mathcal {H}})$, $p(\mathbf {D}|\boldsymbol {\mathcal {H}})$ and $p(\boldsymbol {\mathcal {H}}|\mathbf {D})$ denote the prior distribution, likelihood function and the posterior distribution, respectively, and $Z = \int p(\boldsymbol {\mathcal {H}})Pr(\mathbf {Y}|\boldsymbol {\mathcal {H}}) d\boldsymbol {\mathcal {H}}$, a constant with respect to $\boldsymbol {\mathcal {H}}$, referred to as the evidence. In the SMC framework for static models, rather than obtaining samples directly from the posterior distribution $p(\boldsymbol {\mathcal {H}}|\mathbf {D})$ in (6), a sequence of intermediate target distributions, $\lbrace \pi _{t} \rbrace _{t = 1}^{T}$, are designed, that transitions smoothly from the prior distribution, i.e., $\pi _{1} = p(\boldsymbol {\mathcal {H}})$, which is easier to sample from, and gradually introduces the effect of the likelihood so that in the end, we have $\pi _{T} = p(\boldsymbol {\mathcal {H}}|\mathbf {D})$ which is the posterior distribution of interest [24, 29]. For such sequence of intermediate distributions, a natural choice is the likelihood tempered target sequence [24, 58]:

7 $$ \pi_{t}(\boldsymbol{\mathcal{H}}) = \frac{\Psi_{t}(\boldsymbol{\mathcal{H}})}{Z_{t}} \propto p(\boldsymbol{\mathcal{H}})p(\mathbf{D}|\boldsymbol{\mathcal{H}})^{\epsilon_{t}}  $$

where $\lbrace \epsilon _{t} \rbrace _{t=1}^{T}$ is a non-decreasing temperature schedule with *ε*_1_=0 and *ε*_*T*_=1, $\Psi _{t}(\boldsymbol {\mathcal {H}}) = p(\boldsymbol {\mathcal {H}})p(\boldsymbol {\mathcal {H}}|\mathbf {D})^{\epsilon _{t}}$ is the unnormalized target distribution and $Z_{t} = \int p(\boldsymbol {\mathcal {H}})p(\boldsymbol {\mathcal {H}}|\mathbf {D})^{\epsilon _{t}} d\boldsymbol {\mathcal {H}}$ is the corresponding evidence at time *t*.

Next, we transform this problem in the standard SMC filtering framework [35, 36] by defining a sequence of joint target distributions up to and including time *t*, $\lbrace \tilde {\pi }_{t} \rbrace _{t=1}^{T}$ which admits *π*_*t*_ as marginals as follows:

8 $$ \begin{aligned} \tilde{\pi}_{t}\left(\boldsymbol{\mathcal{H}}_{1:t}\right) &= \frac{\tilde{\Psi}_{t}\left(\boldsymbol{\mathcal{H}}_{1:t}\right)}{Z_{t}} \\ \text{with} ~ \tilde{\Psi}_{t}\left(\boldsymbol{\mathcal{H}}_{1:t}\right) &= \Psi_{t}\left(\boldsymbol{\mathcal{H}}_{t}\right) \prod\limits_{b = 1}^{t-1} \mathcal{L}_{b} \left(\boldsymbol{\mathcal{H}}_{b+1},\boldsymbol{\mathcal{H}}_{b}\right), \end{aligned}  $$

where the artificial kernels $\lbrace \mathcal {L}_{b}\rbrace _{b = 1}^{t-1}$ are referred to as the backward Markov kernels, i.e., $\mathcal {L}_{t}\left (\boldsymbol {\mathcal {H}}_{t+1},\boldsymbol {\mathcal {H}}_{t}\right)$ denotes the probability density of moving back from $\boldsymbol {\mathcal {H}}_{t+1}$ to $\boldsymbol {\mathcal {H}}_{t}$ [24, 29, 59]. Since it is usually difficult to obtain samples directly from the joint target distribution in (8), we define a similar distribution, known as the importance distribution, with a support that includes the support of $\tilde {\pi }_{t}$ [35], from where we can easily draw samples. Following [24, 29, 59], we define the importance distribution $q_{t}\left (\boldsymbol {\mathcal {H}}_{1:t}\right)$ at time *t* as follows:

9 $$ q_{t}(\boldsymbol{\mathcal{H}}_{1:t}) = q_{1}(\boldsymbol{\mathcal{H}}_{1}) \prod\limits_{f = 2}^{t} \mathcal{K}_{f} (\boldsymbol{\mathcal{H}}_{f-1},\boldsymbol{\mathcal{H}}_{f}),  $$

where $ \left \{ \mathcal {K}_{f} \right \}_{f=2}^{t} $ are the Markov transition kernels or forward kernels, i.e., $\mathcal {K}_{t}(\boldsymbol {\mathcal {H}}_{t-1},\boldsymbol {\mathcal {H}}_{t})$ denotes the probability density of moving from $\boldsymbol {\mathcal {H}}_{t-1}$ to $\boldsymbol {\mathcal {H}}_{t}$ [24, 29].

Given that at time *t*−1, we desire to obtain *N* random samples from the target distribution in (8), but as discussed earlier, it is difficult to sample from the target distribution and instead, we obtain the samples from the importance distribution in (9). Following the principle of importance sampling, we then correct for the discrepancy between the target and the importance distributions by calculating the importance weights [35]. The unnormalized weights associated with the *N* samples are obtained as follows:

10 $$ \begin{aligned} &{}\tilde{w}_{t-1}^{n} \!\propto\! \frac{\tilde{\pi}_{t-1}\left(\boldsymbol{\mathcal{H}}_{1:t-1}^{n}\right)}{q_{t-1}\left(\boldsymbol{\mathcal{H}}_{1:t-1}^{n}\right)} \,=\, \frac{\pi_{t-1}\left(\boldsymbol{\mathcal{H}}_{t-1}^{n}\right) \prod_{d = 1}^{t-2} \mathcal{L}_{d} \left(\boldsymbol{\mathcal{H}}_{d+1}^{n},\!\boldsymbol{\mathcal{H}}_{d}^{n}\right)}{q_{1}\left(\boldsymbol{\mathcal{H}}_{1}^{n}\right) \prod_{r = 2}^{t-1} \mathcal{K}_{r} \left(\boldsymbol{\mathcal{H}}_{r-1}^{n},\boldsymbol{\mathcal{H}}_{r}^{n}\right)}\\ &{}\text{and the normalized weights are calculated as:} \\ &{}w_{t-1}^{n} = \frac{\tilde{w}_{t-1}^{n}}{\sum_{l=1}^{N}\tilde{w}_{t-1}^{l}},~ n = 1,...,N. \end{aligned}  $$

As such, the set of weighted samples $\left \{ \boldsymbol {\mathcal {H}}_{1:t-1}^{n}, w_{t-1}^{n} \right \}_{n=1}^{N}$ approximates the joint target distribution $\tilde {\pi }_{t-1}$. To obtain an approximation to the joint target distribution at time *t*, i.e, $\tilde {\pi }_{t}$, the samples are first propagated to the next target distribution $\tilde {\pi }_{t}$ using a forward Markov kernel $\mathcal {K}_{t}(\boldsymbol {\mathcal {H}}_{t-1},\boldsymbol {\mathcal {H}}_{t})$ to obtain the set of particles $\left \{ \boldsymbol {\mathcal {H}}_{1:t}^{n} \right \}_{n = 1}^{N}$. Similar to (10), we then correct for the discrepancy between the importance distribution and the target distribution at time *t*. Thus, the unnormalized weights at time *t* are given as (detail is in the Additional file 1):

11 $$ \begin{aligned} \tilde{w}_{t}^{n} & \propto \tilde{w}_{t-1}^{n}\frac{\Psi_{t}\left(\boldsymbol{\mathcal{H}}_{t}^{n}\right)\mathcal{L}_{t-1}\left(\boldsymbol{\mathcal{H}}_{t}^{n},\boldsymbol{\mathcal{H}}_{t-1}^{n}\right)}{\Psi_{t-1}\left(\boldsymbol{\mathcal{H}}_{t-1}^{n}\right)\mathcal{K}_{t}\left(\boldsymbol{\mathcal{H}}_{t-1}^{n},\boldsymbol{\mathcal{H}}_{t}^{n}\right)} \\ & = \tilde{w}_{t-1}^{n}W_{t}\left(\boldsymbol{\mathcal{H}}_{t-1}^{n},\boldsymbol{\mathcal{H}}_{t}^{n}\right), ~ n = 1,...,N, \end{aligned}  $$

where $\left \{ \tilde {w}_{t-1}^{n} \right \}_{n=1}^{N}$ are the unnormalized weights at time *t*−1, given in (10) and $ \left \{ W_{t}(\boldsymbol {\mathcal {H}}_{t-1}^{n},\boldsymbol {\mathcal {H}}_{t}^{n}) \right \}_{n=1}^{N}$, the unnormalized incremental weights, calculated as:

12 $$ {}W_{t}\left(\boldsymbol{\mathcal{H}}_{t-1}^{n},\boldsymbol{\mathcal{H}}_{t}^{n}\right) \,=\, \frac{\Psi_{t}\left(\boldsymbol{\mathcal{H}}_{t}^{n}\right) \mathcal{L}_{t-1}\left(\boldsymbol{\mathcal{H}}_{t}^{n},\boldsymbol{\theta}_{t-1}^{n}\right)}{\Psi_{t-1}\left(\boldsymbol{\mathcal{H}}_{t-1}^{n}\right) \mathcal{K}_{t}\left(\boldsymbol{\theta}_{t-1}^{n},\boldsymbol{\mathcal{H}}_{t}^{n}\right)}, ~ n = 1,...,N.  $$

According to [29, 60], if a MCMC kernel is considered for the sequence of forward kernel $\lbrace \mathcal {K}_{t}\rbrace _{t=2}^{T}$, then the following $\mathcal {L}_{t}$ is employed:

13 $$ \mathcal{L}_{t-1}(\boldsymbol{\mathcal{H}}_{t},\boldsymbol{\mathcal{H}}_{t-1}) = \frac{\pi_{t}(\boldsymbol{\mathcal{H}}_{t-1})\mathcal{K}_{t}(\boldsymbol{\mathcal{H}}_{t},\boldsymbol{\mathcal{H}}_{t-1})}{\pi_{t}(\boldsymbol{\mathcal{H}}_{t})},  $$

and the unnormalized incremental weights in (12) becomes:

14 $$ W_{t}\left(\boldsymbol{\mathcal{H}}_{t-1}^{n},\boldsymbol{\mathcal{H}}_{t}^{n}\right) = p\left(\mathbf{D}|\boldsymbol{\mathcal{H}}_{t-1}^{n}\right)^{(\epsilon_{t} - \epsilon_{t-1})},~ n = 1,...,N,  $$

(detail is in the Additional file 1) where *ε*_*t*_−*ε*_*t*−1_ is the step length of the cooling schedule of the likelihood at time *t*. Note that $p\left (\mathbf {D}|\boldsymbol {\mathcal {H}}_{t-1}^{n}\right), n = 1,...,N$ can easily be computed as highlighted in [53].

However, in the SMC procedure described above, after some iterations, all samples except one will have very small weights, a phenomenon referred to as degeneracy in the literature. It is unavoidable as it has been shown that the variance of the importance weights increases over time [35]. An adaptive way to check this is by computing the effective sample size (ESS) as:

15 $$ ESS = \frac{1}{\sum_{n=1}^{N} \left(w_{t}^{n} \right)^{2}}.  $$

Details on when to resample and the resampling procedure are in the Additional file 1.

Finally, the SMC algorithm for the estimation of *Θ* is presented in Algorithm 1. In the algorithm, $p(\boldsymbol {\mathcal {H}}) = p(\boldsymbol {\lambda })p(\Theta)p(\Upsilon |\Theta)$ and $Pr(\mathbf {D}|\boldsymbol {\mathcal {H}}) = Pr(\mathbf {D}|\Upsilon,\boldsymbol {\lambda })$ which can easily be computed using the procedures highlighted in [53]. Similarly, *p*(*Υ*|*Θ*) can be calculated with the expression in (2), *p*(*Θ*)=1/*Θ*_max_ and *p*(***λ***) is calculated from the assumed standard prior distribution(s) for the elements in ***λ***. For the details of the different mutational models, their respective parameter(s) and the assumed prior distribution(s), please see the Additional file 1. Also in Algorithm 1, *V* denotes the number of parameters, including the genealogy and *R*_*MCMC*_ denotes the chain length for each particle. In lines 17 and 18 of Algorithm 1, the *π*_*t*_ invariant Markov kernel is described in Algorithm 2 in the Additional file 1.



## Results

In this section, we demonstrate the performance of the proposed SMC algorithm using both simulated datasets and real biological sequences. In addition, we compare the estimates obtained from the proposed SMC algorithm to that of the MH-MCMC algorithm. In the experiments with MH-MCMC (details in [61]), we set the burn-in period to 50000 iterations and the chain length to 20000 iterations to approximate the posterior estimates.

### Simulated data

Simulated datasets were generated from the programs *ms* [46] and *Seq-Gen* [47]. With the *Seq-Gen* program, we were able to generate sequences under a variety of finite-site models. Specifically, *ms* is used to generate possible tree structure and the resulting tree structure is given as an input into the *Seq-Gen* program, and DNA sequences are generated under an appropriate finite-site model. DNA sequences were generated with varying values of *Θ*, number of sequences sampled (*m*), length of sequence in each sample (*l*), and mutational model (specific values are shown in Table 1). For each combination of *Θ*, *m*, and *l* under a mutation model, we evaluate the proposed SMC algorithm and the MH-MCMC for the generated data.

**Table 1:** Estimates of the mean and standard deviation of *Θ* obtained from the two methods with the K80 model

	*m* = 20							
	*l* =200			*l* = 400			*l* = 600	
*Θ*	SMC	MCMC		SMC	MCMC		SMC	MCMC
0.01	0.0081 (0.0036)	0.0009 (0.0053)		0.0113 (0.0030)	0.0010 (0.0051)		0.0101 (0.0025)	0.0096 (0.0045)
0.10	0.0795 (0.0040)	0.0193 (0.0050)		0.0881 (0.0040)	0.0280 (0.0045)		0.1121 (0.0034)	0.0924 (0.0042)
0.50	0.4023 (0.0044)	0.3034 (0.0050)		0.4412 (0.0044)	0.4214 (0.0049)		0.4624 (0.0039)	0.4510 (0.0040)

*m*=20 and *l*=200,400 and 600. The different values of *Θ* are shown in column 1

**Fig. 2:**
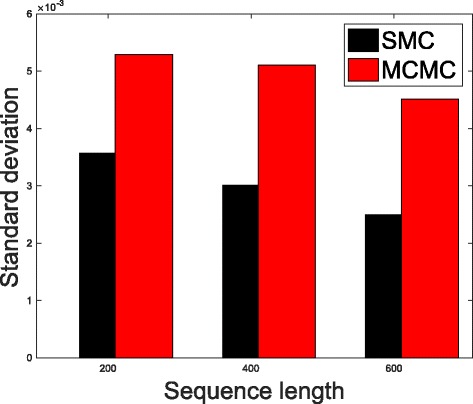
Plot of standard deviation. Plot of standard deviation versus sequence length (*l*) for the two methods. Sample size, *m*=20, *Θ*=0.01 and the model of evolution is K80

**Fig. 3:**
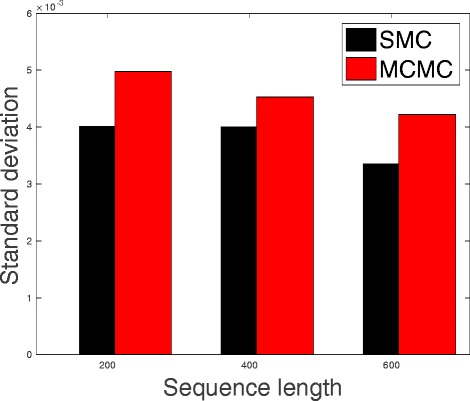
Plot of standard deviation. Plot of standard deviation versus sequence length (*l*) for the two methods. Sample size, *m*=20, *Θ*=0.1 and the model of evolution is K80

**Fig. 4:**
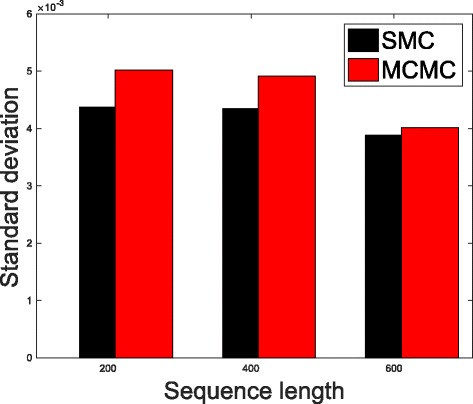
Plot of standard deviation. Plot of standard deviation versus sequence length (*l*) for the two methods. Sample size, *m*=20, *Θ*=0.5 and the model of evolution is K80

**Fig. 5:**
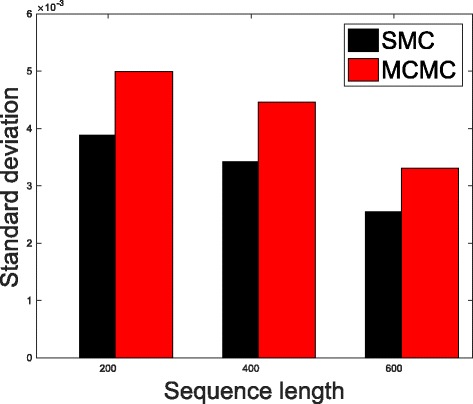
Plot of standard deviation. Plot of standard deviation versus sequence length (*l*) for the two methods. Sample size, *m*=20, *Θ*=0.01 and the model of evolution is F84

**Fig. 6:**
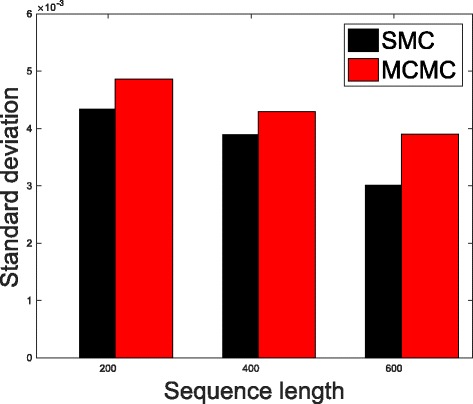
Plot of standard deviation. Plot of standard deviation versus sequence length (*l*) for the two methods. Sample size, *m*=20, *Θ*=0.1 and the model of evolution is F84

**Fig. 7:**
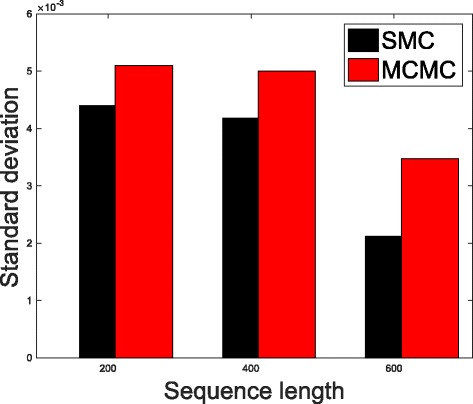
Plot of standard deviation. Plot of standard deviation versus sequence length (*l*) for the two methods. Sample size, *m*=20, *Θ*=0.5 and the model of evolution is F84

**Fig. 8:**
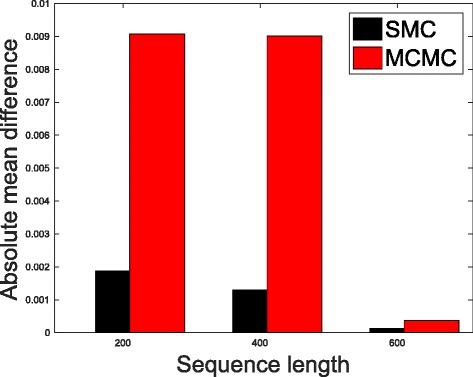
Plot of absolute differences. Sample size, *m*=20, *Θ*=0.01 and the model of evolution is K80

**Fig. 9:**
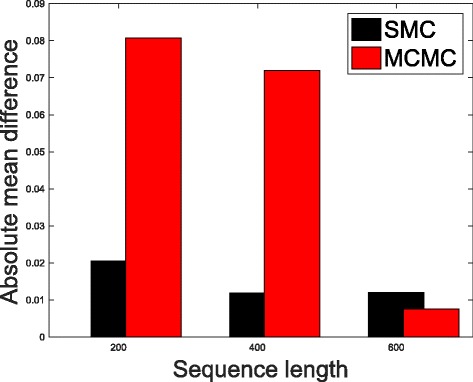
Plot of absolute differences. Sample size, *m*=20, *Θ*=0.1 and the model of evolution is K80

**Fig. 10:**
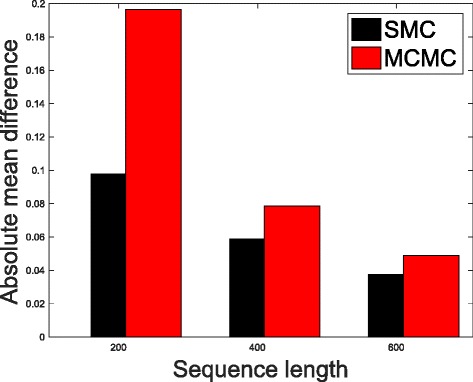
Plot of absolute differences. Sample size, *m*=20, *Θ*=0.5 and the model of evolution is K80

**Fig. 11:**
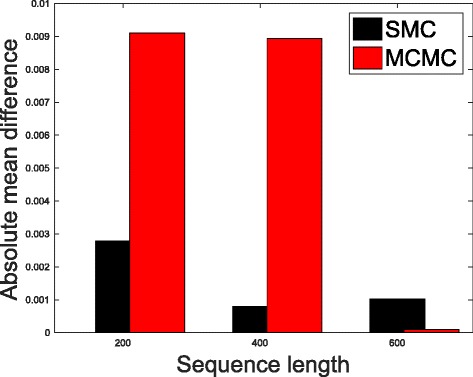
Plot of absolute differences. Sample size, *m*=20, *Θ*=0.01 and the model of evolution is F84

**Fig. 12:**
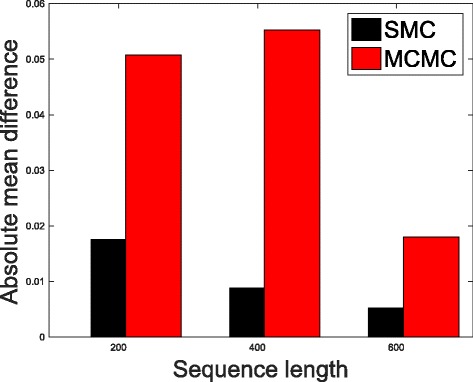
Plot of absolute differences. Sample size, *m*=20, *Θ*=0.1 and the model of evolution is F84

**Fig. 13:**
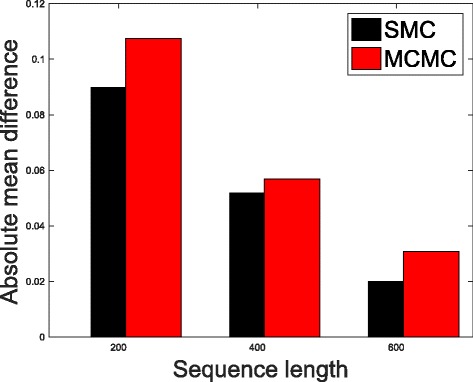
Plot of absolute differences. Sample size, *m*=20, *Θ*=0.5 and the model of evolution is F84

In Table 1, we present the results obtained from the datasets generated from the two-parameter K80 model of evolution [54]. The results in Table 1 show the true value of *Θ* used in generating the sequence data, the number of sequences sampled (*m*), the length of each sequence in a particular sample (*l*) and the chosen model of evolution. The estimated mean values of *Θ* obtained from each of the methods are shown directly under the method, and the standard deviation is shown next to the mean value in parenthesis. Largely, the methods returned mean estimates that are close to the true values of *θ*. However, the SMC algorithm produced smaller standard deviation on almost all the datasets. This is not so surprising because after the particles have been resampled, those with smaller weights are often discarded and would eventually be replaced by the ones with relatively larger weights, these are the particles that better explain the observed data as the algorithm progresses. To further consolidate the results obtained with the K80 model, we present the results obtained from the two methods with the data generated with the F84 [55] model. Similar trends are observed in all our experiments and the comprehensive results are presented in Table 2. In Figs. 2, 3, 4, 5, 6, and 7, the pictorial view of how the standard deviation changes as the length of sequences increases is presented and similarly, in Figs. 8, 9, 10, 11, 12, and 13, the absolute difference between the true mean and the estimated mean is plotted as a function of sequence length, *l*.

**Table 2:** Estimates of the mean and standard deviation of *Θ* obtained from the two methods with the F84 model

	*m* = 20							
	*l* =200			*l* = 400			*l* = 600	
*Θ*	SMC	MCMC		SMC	MCMC		SMC	MCMC
0.01	0.0072 (0.0039)	0.0009 (0.0050)		0.0108 (0.0034)	0.0011 (0.0045)		0.0110 (0.0026)	0.0099 (0.0033)
0.10	0.0824 (0.0043)	0.0493 (0.0049)		0.0911 (0.0039)	0.0448 (0.0043)		0.1052 (0.0030)	0.0820 (0.0039)
0.50	0.4101 (0.0044)	0.3926 (0.0051)		0.4482 (0.0042)	0.4431 (0.0050)		0.4800 (0.0021)	0.4692 (0.0035)

*m*=20 and *l*=200,400 and 600. The different values of *Θ* are shown in column 1

### Mitochondrial DNA sequence data (mtDNA)

We next evaluate our algorithm on the Mitochondrial DNA sequence dataset [48]. This dataset contains 360 bp from the mitochondrial control region of 63 Amerindians of the Nuu-Chah-Nulth tribe [45]. In analyzing this particular dataset, we assumed the F84 model. With this assumption, it means that the nucleotide frequency, ***π*** and the transition-transversion ratio, *κ* will also be estimated alongside *Θ*. One important observation with this dataset is that the mtDNA is haploid and maternally inherited. Hence, *Θ*=2*N*_*f*_ *μ* where *N*_*f*_ is the number of females. The full dataset was analyzed with the proposed SMC method and the MCMC algorithm. The estimated mean of *Θ* 0.0451 obtained from the proposed SMC method is slightly higher than 0.0402 that was recorded for the MCMC-based algorithm. Although, the true value of *Θ* is not available for this dataset, we can draw some inference from the values of standard deviation from both methods. The proposed SMC algorithm produced a slightly smaller standard deviation (0.00327) compared to its MCMC-based counterpart (0.00866).

## Discussion

In this paper, we considered the problem of estimating the scaled mutation rate, *Θ* from samples of molecular sequence data. We present a novel Bayesian approach based on the SMC algorithm for static models which samples from the joint distribution of *Θ*, the genealogy and the unknown parameters of the mutational model. Specifically, the unknown genealogy relating the sampled sequences is considered as one of the unknown parameters in the Bayesian setup. Although, the space of the possible genealogies that describe the dataset is infinitely large, the algorithm is implemented in such a way that only the highly probable samples from the posterior distribution of the genealogy are considered in estimating the parameters of interest. Hence, the marginal distribution of the parameter of interest, *Θ* is approximated from the joint posterior distribution of all the parameters by a set of weighted samples.

We have performed series of experiments on simulated datasets (varying the true value of *Θ*, the sequence length *l*, the number of sampled sequences from the population *m* and the mutational model) and real biological sequences to evaluate the performance of the proposed SMC algorithm. With all the experiments run and the results obtained, we have shown that the SMC algorithm for static model is a promising alternative to the standard MCMC methods to simulate from the static target distributions of the parameters of population genetics models based on the coalescent. The parameters of the proposed SMC algorithm, i.e. the number of particles and iterations are set in such a way that on the average, both algorithms have equal runtimes. However, since the proposed SMC algorithm can be parallelized when the resources are available, this can tremendously lower its runtime and as such, increases its efficiency.

In the proposed SMC algorithm, the experiments are initialized by taking samples from the prior distributions of the unknown parameters in the Bayesian setup. In the case of the genealogy, we first applied the Unweighted Pair Group Method with Arithmetic Mean (UPGMA) phylogeny reconstruction algorithm to the sequence data and used the resulting tree to guide the sampling procedure from the prior distribution of the genealogy. We noticed that doing this dramatically reduced the number of samples (*N*=500) needed to obtained a good approximation to the posterior distribution of *Θ*.

## Conclusions

Finally, we have demonstrated the efficacy of the proposed algorithm with the DNA sequence data, using various models of evolution with a single population. However, this algorithm can also be used to analyze other types of data, ranging from the RNA sequence data, protein sequence data, microsatellite data, etc., inasmuch as the appropriate model is specified. Every details of the algorithm remains the same except for the calculation of the likelihood function as a result of the model change. In addition, the current work can potentially be extended to cases involving varying population size, migration, and recombination which all involve more complex models of population.

## Additional file

Additional file 1 Supplementary Material. (PDF 232 kb)

## Abbreviations

ABC: Approximate Bayesian computation
DNA: Deoxyribonucleic acid
ESS: Effective sample size
IS: Importance sampling
MCMC: Markov Chain Monte Carlo
MH-MCMC: Metropolis-hastings MCMC
MRCA: Most recent common ancestor
mtDNA: Mitochondrial DNA
PDF: Probability density function
RNA: Ribonucleic acid
SMC: Sequential Monte Carlo
UPGMA: Unweighted pair group method with arithmetic mean
