# Supplementary material for: Bayesian estimation of scaled mutation rate under the coalescent: a sequential Monte Carlo approach
Source: BMC Bioinformatics. 2017 Dec 8;18:541. doi: 10.1186/s12859-017-1948-6 (PMC5721689; doi:10.1186/s12859-017-1948-6)
Supplement: Supplementary file 1 — Supplementary Material. (PDF 232 kb) [file 12859_2017_1948_MOESM1_ESM.pdf]

Supplemental Material for “Bayesian Estimation of Scaled Mutation Rate Under the Coalescent: A Sequential Monte Carlo Approach”

Oyetunji Ogundijo [1] and Xiaodong Wang [1,*]

Note S2

In our analyses of datasets, we considered two different mutational models, namely: K80 and F84 models.

K80 model:

Kimura’s two-parameter, K80 model allows for two parameters, different rates for transitions (changes involving purine-to-purine or pyrimidine-to-pyrimidine) and transversions (purine-to-pyrimidine and vice-versa) i.e., it distinguishes between the rate of transitions and transversions using the κ parameter. In addition, the nucleotide frequencies are the same i.e $\pi_A = \pi_C = \pi_G = \pi_T = 0.25$. The three types of changes are:

$$\begin{aligned} P(t)_{ii} &= \frac{1}{4} + \frac{3}{4}e^{-\mu t}, \\ P(t)_{ij,\text{Transition}} &= \frac{1}{4} + \frac{1}{4}e^{-\mu t} + \frac{1}{2}e^{-\mu t\left(\frac{\kappa+1}{2}\right)}, \\ P(t)_{ij,\text{Transversion}} &= \frac{1}{4} - \frac{1}{4}e^{-\mu t}, \end{aligned}$$

where $P(t)_{ii}$ is the probability of a nucleotide to remain the same during the evolutionary time t and $P(t)_{ij}$ is the probability of a nucleotide changing from initial state i to final state j during the evolutionary time t.

*to whom correspondence should be addressed

F84 model:

F84 model distinguishes between the rate of transitions and transversions using the κ parameter and in addition, it allows unequal nucleotide frequencies i.e., $\pi_A \neq \pi_C \neq \pi_G \neq \pi_T$. Thus, both κ and the nucleotide frequencies are to be estimated when F84 model is considered. The three types of changes are:

$$
\begin{aligned}
P(t)_{jj} &= \pi_j + \pi_j\Big(\frac{1}{\Pi_j} - 1\Big)e^{-\mu t} + \Big(\frac{\Pi_j - \pi_j}{\Pi_j} - 1\Big)e^{-\mu t(\kappa+1)},\\
P(t)_{ij,\text{Transition}} &= \pi_j + \pi_j\Big(\frac{1}{\Pi_j} - 1\Big)e^{-\mu t} - \Big(\frac{\pi_j}{\Pi_j} - 1\Big)e^{-\mu t(\kappa+1)},\\
P(t)_{ij,\text{Transversion}} &= \pi_j(1 - e^{-\mu t}),
\end{aligned}
$$

where $\Pi_j = \pi_A + \pi_G$ if j is a purine (A or G) and $\Pi_j = \pi_C + \pi_T$ if j is a pyrimidine (C or T).

Note S3

Here, we present the prior distributions for all the unknown parameters in the Bayesian model that we considered.

Prior density of Θ:

Θ is a positive parameter and we impose a uniform distribution in the interval $[0, \Theta_{\max}]$,i.e.,

$$\Theta \sim \mathcal{U}(0, \Theta_{\max}),$$

with the probability density function (PDF) given as

$$p(\Theta) = \begin{cases} \frac{1}{\Theta_{\max}}, & \text{for } \Theta \in [0, \Theta_{\max}] \\ 0, & \text{otherwise.} \end{cases}$$

Table 1 shows the values of $\Theta_{\max}$ considered in the experiments with simulated datasets and the mitochondrial DNA sequence data (mtDNA data). When analyzing real biological datasets, results obtained from some other estimation approaches can provide knowledge about the range of $\Theta_{\max}$.

	$\Theta_{\max}$ for the prior distribution
Simulated data: $\Theta = 0.01$	0.10
Simulated data: $\Theta = 0.10$	1.00
Simulated data: $\Theta = 0.50$	1.00
mtDNA data	1.00

Table 1: Chosen values of $\Theta_{\max}$ for all datasets.

Prior density of $\boldsymbol{\lambda}$:

$\boldsymbol{\lambda}$ is the set of unknown parameters associated with each mutational model and as stated earlier, the mutational models considered are K80 and F84 models. K80 model has only one unknown parameter i.e., the transition-transversion ratio κ while the F80 model has two unknown parameters i.e., κ and the nucleotide frequencies $\boldsymbol{\pi}$.

Prior density of κ:

Like Θ, κ is a positive parameter and we impose a uniform distribution in the interval $[0, \kappa_{\max}]$,i.e.,

$$\kappa \sim \mathcal{U}(0, \kappa_{\max}),$$

and the PDF is given as

$$p(\kappa) = \begin{cases} \frac{1}{\kappa_{\max}}, & \text{for } \kappa \in [0, \kappa_{\max}] \\ 0, & \text{otherwise.} \end{cases}$$

Table 2 shows the values of $\kappa_{\max}$ considered in the experiments with simulated datasets and the mtDNA dataset.

	$\kappa_{\max}$ for the prior distribution
Simulated data: $\kappa = 2$	10
mtDNA data	200

Table 2: In simulated datasets, true $\kappa = 2$ and $\kappa_{\max} = 10$.

Prior density of $\boldsymbol{\pi}$:

Here, we impose Dirichlet distribution on the nucleotide frequencies $\boldsymbol{\pi}$ i.e.,

$$\boldsymbol{\pi} \sim \mathrm{Dir}(\boldsymbol{\alpha}),$$

and the PDF is given by

$$p(\boldsymbol{\pi}|\boldsymbol{\alpha}) = \frac{\Gamma(\sum_i \alpha_i)}{\prod_i \Gamma(\alpha_i)} \prod_i \pi_i^{\alpha_i - 1}, \quad i \in \{A, C, G, T\},$$

where $\Gamma(\cdot)$ is the gamma function, $\boldsymbol{\alpha} = (\alpha_A, \alpha_C, \alpha_G, \alpha_T)$ are the concentration parameters and $\pi_A + \pi_C + \pi_G + \pi_T = 1$. In our experiments, we chose $\boldsymbol{\alpha} = \beta_0 \boldsymbol{\pi}_{\mathrm{emp}}$, where $\boldsymbol{\pi}_{\mathrm{emp}}$ is the empirical value of the nucleotide frequencies calculated from the data and β_0, a scalar value, chosen to be 10, is used to control the peakedness of the prior distribution of $\boldsymbol{\pi}$ around $\boldsymbol{\pi}_{\mathrm{emp}}$ [1].

Prior density of the genealogy:

Algorithm 1 describes the procedure to sample from the distribution of the possible genealogies of m copies in a population with effective population size N_e.

Algorithm 1 The coalescent: Simulation of a random genealogy.

1: Start with m lineages.
2: Set $k = m$ and $T = 0$.
3: Draw a random quantity t_k from an exponential distribution with expectation $4N_e/(k(k-1))$.
4: Randomly pick two of the k copies of the gene, without replacement.
5: Create a node of the genealogical tree which is the immediate common ancestor of these two copies, and which existed $T + t_k$ generations before the present.
6: Set $T = T + t_k$.
7: Replace these two copies by this common ancestor and set $k = k - 1$.
8: If $k = 1$ we are done. Otherwise return to step 3.

Note S4

Obtaining the weight at time t from the weights at time t-1

In the main text, it was shown that the set of weighted samples $\{\boldsymbol{\mathcal{H}}^n_{1:t-1}, w^n_{t-1}\}^N_{n=1}$ approximates the joint target distribution $\tilde{\pi}_{t-1}$. After propagating the sam-

ples at time $t-1$ using a forward Markov kernel $\mathcal{K}_t(\boldsymbol{\mathcal{H}}_{t-1}, \boldsymbol{\mathcal{H}}_t)$, the unnormalized weights at time t are calculated as follows:

$$\begin{aligned}
\tilde{w}_t^n &\propto \frac{\tilde{\pi}_t(\boldsymbol{\mathcal{H}}_{1:t}^n)}{q_t(\boldsymbol{\mathcal{H}}_{1:t}^n)} \\
&= \frac{\pi_t(\boldsymbol{\mathcal{H}}_t^n)\prod_{d=1}^{t-1}\mathcal{L}_d(\boldsymbol{\mathcal{H}}_{d+1}^n, \boldsymbol{\mathcal{H}}_d^n)}{q_1(\boldsymbol{\mathcal{H}}_1^n)\prod_{r=2}^{t}\mathcal{K}_r(\boldsymbol{\mathcal{H}}_{r-1}^n, \boldsymbol{\mathcal{H}}_r^n)} \\
&= \frac{\pi_t(\boldsymbol{\mathcal{H}}_t^n)\mathcal{L}_{t-1}(\boldsymbol{\mathcal{H}}_t^n, \boldsymbol{\mathcal{H}}_{t-1}^n)\prod_{d=1}^{t-2}\mathcal{L}_d(\boldsymbol{\mathcal{H}}_{d+1}^n, \boldsymbol{\mathcal{H}}_d^n)}{q_1(\boldsymbol{\mathcal{H}}_1^n)\mathcal{K}_t(\boldsymbol{\mathcal{H}}_{t-1}^n, \boldsymbol{\mathcal{H}}_t^n)\prod_{r=2}^{t-1}\mathcal{K}_r(\boldsymbol{\mathcal{H}}_{r-1}^n, \boldsymbol{\mathcal{H}}_r^n)} \\
&= \frac{\pi_t(\boldsymbol{\mathcal{H}}_t^n)\mathcal{L}_{t-1}(\boldsymbol{\mathcal{H}}_t^n, \boldsymbol{\mathcal{H}}_{t-1}^n)\pi_{t-1}(\boldsymbol{\mathcal{H}}_{t-1}^n)\prod_{d=1}^{t-2}\mathcal{L}_d(\boldsymbol{\mathcal{H}}_{d+1}^n, \boldsymbol{\mathcal{H}}_d^n)}{\pi_{t-1}(\boldsymbol{\mathcal{H}}_{t-1}^n)\mathcal{K}_t(\boldsymbol{\mathcal{H}}_{t-1}^n, \boldsymbol{\mathcal{H}}_t^n)q_1(\boldsymbol{\mathcal{H}}_1^n)\prod_{r=2}^{t-1}\mathcal{K}_r(\boldsymbol{\mathcal{H}}_{r-1}^n, \boldsymbol{\mathcal{H}}_r^n)} \\
\tilde{w}_t^n &\propto \tilde{w}_{t-1}^n \frac{\pi_t(\boldsymbol{\mathcal{H}}_t^n)\mathcal{L}_{t-1}(\boldsymbol{\mathcal{H}}_t^n, \boldsymbol{\mathcal{H}}_{t-1}^n)}{\pi_{t-1}(\boldsymbol{\mathcal{H}}_{t-1}^n)\mathcal{K}_t(\boldsymbol{\mathcal{H}}_{t-1}^n, \boldsymbol{\mathcal{H}}_t^n)},
\end{aligned}$$

from the definitions of π_t and π_{t-1} in the main text and noticing that Z_t and Z_{t-1} are constants with respect to $\boldsymbol{\mathcal{H}}_t^n$ and $\boldsymbol{\mathcal{H}}_{t-1}^n$, then

$$\begin{aligned}
\tilde{w}_t^n &\propto \tilde{w}_{t-1}^n \frac{\Psi_t(\boldsymbol{\mathcal{H}}_t^n)\mathcal{L}_{t-1}(\boldsymbol{\mathcal{H}}_t^n, \boldsymbol{\mathcal{H}}_{t-1}^n)}{\Psi_{t-1}(\boldsymbol{\mathcal{H}}_{t-1}^n)\mathcal{K}_t(\boldsymbol{\mathcal{H}}_{t-1}^n, \boldsymbol{\mathcal{H}}_t^n)} \\
&= \tilde{w}_{t-1}^n W_t(\boldsymbol{\mathcal{H}}_{t-1}^n, \boldsymbol{\mathcal{H}}_t^n), \;\; n = 1, ..., N,
\end{aligned}$$

where $\{\tilde{w}_{t-1}^n\}_{n=1}^N$ are the unnormalized weights at time $t-1$ and $\{W_t(\boldsymbol{\mathcal{H}}_{t-1}^n, \boldsymbol{\mathcal{H}}_t^n)\}_{n=1}^N$, the unnormalized incremental weights, calculated as

$$W_t(\boldsymbol{\mathcal{H}}_{t-1}^n, \boldsymbol{\mathcal{H}}_t^n) = \frac{\Psi_t(\boldsymbol{\mathcal{H}}_t^n)\mathcal{L}_{t-1}(\boldsymbol{\mathcal{H}}_t^n, \boldsymbol{\theta}_{t-1}^n)}{\Psi_{t-1}(\boldsymbol{\mathcal{H}}_{t-1}^n)\mathcal{K}_t(\boldsymbol{\theta}_{t-1}^n, \boldsymbol{\mathcal{H}}_t^n)}, \; n = 1, ..., N.$$

Target Distributions, Forward and Backward Kernels Specification Population Parameters Estimation

Here, we specify the exact form of the sequence of target distributions $\{\pi_t\}_{t=1}^T$, the forward kernels, $\{\mathcal{K}_t\}_{t=2}^T$ and the backward kernels $\{\mathcal{L}_{t-1}\}_{t=2}^T$.

Sequence of target distributions and forward kernels:

Since we are interested in the likelihood tempered target sequence we sample initially from the prior distribution $\pi_1 = p(\boldsymbol{\mathcal{H}})$ and gradually introduce the effect of the likelihood in order to obtain an approximation of the posterior distribution $p(\boldsymbol{\mathcal{H}}|\mathbf{D})$, i.e, π_T at $t = T$. Although, there are a few choices of $\mathcal{K}_t$ that have been proposed, we employ MCMC kernels of invariant distribution

π_t, as $\mathcal{K}_t$. There is more freedom in specifying the forward kernels in SMC since kernels do not need to be reversible or even Markov. As such, we will employ a hybrid Gibbs and Metropolis-Hastings (M-H) samplers that allows local moves in order to successively move all the parameters of interest in $\boldsymbol{\mathcal{H}}$ i.e., Θ, Υ and $\boldsymbol{\lambda}$. The set of proposal distributions for the genealogy and other parameters are discussed below.

Sequence of backward kernels:

To obtain a good performance of the SMC samplers algorithm, the backward kernel $\mathcal{L}_t$ is often optimized with respect to the forward kernel $\mathcal{K}_t$. Thus, if an MCMC kernel is used as forward kernel, then the following $\mathcal{L}_t$ is employed:

$$\mathcal{L}_{t-1}(\boldsymbol{\mathcal{H}}_t, \boldsymbol{\mathcal{H}}_{t-1}) = \frac{\pi_t(\boldsymbol{\mathcal{H}}_{t-1})\mathcal{K}_t(\boldsymbol{\mathcal{H}}_t, \boldsymbol{\mathcal{H}}_{t-1})}{\pi_t(\boldsymbol{\mathcal{H}}_t)},$$

a good approximation of the optimal backward kernel when the discrepancy between π_t and π_{t-1} is small. Thus, since the chosen $\mathcal{L}_t$ is the reversal Markov kernel associated with $\mathcal{K}_t$, then the unnormalized incremental weights become :

$$\begin{aligned} W_t(\boldsymbol{\mathcal{H}}_{t-1}^n, \boldsymbol{\mathcal{H}}_t^n) &= \frac{\Psi_t(\boldsymbol{\mathcal{H}}_t^n)\pi_t(\boldsymbol{\mathcal{H}}_{t-1}^n)}{\Psi_{t-1}(\boldsymbol{\mathcal{H}}_{t-1}^n)\pi_t(\boldsymbol{\mathcal{H}}_t^n)} \\ &= \frac{p(\boldsymbol{\mathcal{H}}_t^n)p(\mathbf{D}|\boldsymbol{\mathcal{H}}_t^n)^{\epsilon_t}p(\boldsymbol{\mathcal{H}}_{t-1}^n)p(\mathbf{D}|\boldsymbol{\mathcal{H}}_{t-1}^n)^{\epsilon_t}}{p(\boldsymbol{\mathcal{H}}_{t-1}^n)p(\mathbf{D}|\boldsymbol{\mathcal{H}}_{t-1}^n)^{\epsilon_{t-1}}p(\boldsymbol{\mathcal{H}}_t^n)p(\mathbf{D}|\boldsymbol{\mathcal{H}}_t^n)^{\epsilon_t}} \\ &= p(\mathbf{D}|\boldsymbol{\mathcal{H}}_{t-1}^n)^{(\epsilon_t-\epsilon_{t-1})}, \;\; n = 1, ..., N. \end{aligned}$$

Resampling Procedure

Resampling is performed when the ESS is significantly less than the number of samples, discarding the ineffective samples and then multiply the effective ones. In all our experiments, we performed resampling when the ESS is less than $N/10$. The resampling procedure is briefly summarized as follows:

- Interpret each weight w_t^n as the probability of obtaining the sample index n in the set $\{\boldsymbol{\mathcal{H}}_t^n : n = 1, ..., N\}$.
- Draw N samples from the discrete probability distribution and replace the old sample set with this new one.
- Set all weights to the constant value $w_t^n = 1/N$.

Note S5

Here, we present the hybrid Gibbs and Metropolis-Hastings algorithm of invariant distribution π_t employed to move the particles in the SMC algorithm presented in the main text. In the algorithm, V is the total number of parameters and R_{MCMC} is the chain length for each sample (particle).

Algorithm 2 Hybrid Gibbs and M-H Kernel $\mathcal{K}(\cdot;\cdot)$ for the n-th sample

Initialization Set $\boldsymbol{\mathcal{H}}^0 = [\sigma_1^0, ..., \sigma_V^0] = \boldsymbol{\mathcal{H}}_{t-1}^n = [\sigma_{1,t-1}^n, ..., \sigma_{V,t-1}^n]$
for $r = 1, ..., R_{MCMC}$ **do**
  for $v = 1, ..., V$ **do**
    (a) Sample $\sigma_v^* \sim h_v(\sigma_v^*|\sigma_v^{r-1})$, where $h_v(\cdot|\cdot)$ is the proposal distribution for the vth parameter (See below for details).
    (b) Compute the acceptance ratio:
    $$\alpha = \min\left\{1, \frac{Pr(\mathbf{D}|\boldsymbol{\mathcal{H}}^*)^{\epsilon_t} p(\boldsymbol{\mathcal{H}}^*)}{Pr(\mathbf{D}|\boldsymbol{\mathcal{H}}^{r-1})^{\epsilon_t} p(\boldsymbol{\mathcal{H}}^{r-1})} \frac{h_v(\sigma_v^{r-1}|\sigma_v^*)}{h_v(\sigma_v^*|\sigma_v^{r-1})}\right\},$$
      where $\boldsymbol{\mathcal{H}}^* = [\sigma_1^r, ..., \sigma_{v-1}^r, \sigma_v^*, \sigma_{v+1}^{r-1}, ..., \sigma_V^{r-1}]$,
      and $\boldsymbol{\mathcal{H}}^{r-1} = [\sigma_1^r, ..., \sigma_{v-1}^r, \sigma_v^{r-1}, \sigma_{v+1}^{r-1}, ..., \sigma_V^{r-1}]$
    (c) Sample a random variate $u \sim \mathcal{U}(0, 1)$
    if $u \leqslant \alpha$ **then**
      $\sigma_v^r = \sigma_v^*$
    else
      $\sigma_v^r = \sigma_v^{r-1}$
    end if
  end for
end for
Set the new particle value at time t as $\boldsymbol{\mathcal{H}}_t^n = [\sigma_1^{R_{MCMC}}, ..., \sigma_V^{R_{MCMC}}]$.

Proposal distributions:

Next, we present the proposal distributions $h_v(\cdot|\cdot)$ in **Algorithm 2** for all the parameters in $\boldsymbol{\mathcal{H}}$.

Proposal distribution for Θ:

The update algorithm here is similar to the one described in [2]. Let the previous value in the Markov chain be Θ_{r-1}; a proposal value, Θ^* is drawn

from a uniform distribution in the interval $[\Theta_{r-1} - \Delta_\Theta, \Theta_{r-1} + \Delta_\Theta]$, i.e.,

$$\Theta^* \sim \mathcal{U}(\Theta_{r-1} - \Delta_\Theta, \Theta_{r-1} + \Delta_\Theta),$$

where Δ_Θ is chosen such that $\Delta_\Theta < \Theta_{\max}$ (note: $\Theta_{\max}$ is defined in the prior distribution.). Thus, if $\Theta^* < 0$, set $\Theta^* = -\Theta^*$, and if $\Theta^* > \Theta_{\max}$, set $\Theta^* = 2\Theta_{\max} - \Theta^*$.

Proposal distribution for κ:

Similar to the update algorithm for Θ, let the previous value in the Markov chain be κ_{r-1}; a proposal value, κ^* is drawn from a uniform distribution in the interval $[\kappa_{r-1} - \Delta_\kappa, \kappa_{r-1} + \Delta_\kappa]$, i.e.,

$$\kappa^* \sim \mathcal{U}(\kappa_{r-1} - \Delta_\kappa, \kappa_{r-1} + \Delta_\kappa),$$

where Δ_κ is chosen such that $\Delta_\kappa < \kappa_{\max}$ ($\kappa_{\max}$ is defined in the prior distribution for κ). Therefore, if $\kappa^* < 0$, set $\kappa^* = -\kappa^*$, and if $\kappa^* > \kappa_{\max}$, set $\kappa^* = 2\kappa_{\max} - \kappa^*$.

Proposal distribution for $\boldsymbol{\pi}$:

Given that the previous value of $\boldsymbol{\pi}$ in the Markov chain is denoted by $\boldsymbol{\pi}_{r-1}$, a proposal value $\boldsymbol{\pi}^*$ is drawn as follows:

$$\boldsymbol{\pi}^* \sim \mathrm{Dir}(\beta_1 \boldsymbol{\pi}_{r-1}),$$

$\mathrm{Dir}(\cdot)$ is a Dirichlet distribution with concentration parameters given as $\beta_1 \boldsymbol{\pi}_{r-1} = (\beta_1 \pi_{A,r-1}, \beta_1 \pi_{C,r-1}, \beta_2 \pi_{G,r-1}, \beta_1 \pi_{T,r-1})$. Similar to β_0, β_1 is used to control the peakedness of the transition kernel around the previously sampled value $\boldsymbol{\pi}_{r-1}$. $\beta_1 = 100$ in all the experiments.

Proposal distribution for genealogy:

The conditional coalescent proposal for the genealogy is well described in [2, 3, 4] and the algorithms can easily be implemented.

References

[1] Erkkilä,T., et al. (2010) Probabilistic analysis of gene expression measurements from heterogeneous tissues. *Bioinformatics*, **26**, 2571–2577.

[2] Nielsen,R., Wakeley,J. (2001) Distinguishing migration from isolation: a Markov chain Monte Carlo approach. *Genetics*, **158**, 885–896.

[3] Felsenstein,J., et al. (1999) Likelihoods on coalescents: a Monte Carlo sampling approach to inferring parameters from population samples of molecular data. *Lecture Notes-Monograph Series*, 163–185.

[4] Stephens,M. (2001) Inference under the coalescent. *Handbook of statistical genetics.*
